# Effects of Malic Acid on Cadmium Uptake and Translocation and Essential Element Accumulation in Rice

**DOI:** 10.3390/toxics13100811

**Published:** 2025-09-24

**Authors:** Shuo Zhang, Yiteng Zhang, Guoyi Lv, Tianqi Liu, Zhongqi Liu, Yubo Jiang, Yubo Hao, Yang Yu, Wenjun Dong, Chunrong Qian

**Affiliations:** 1 Heilongjiang Academy of Agricultural Sciences, Harbin 150086, China; shuozhang994@163.com (S.Z.); yiteng_fighting@163.com (Y.Z.); lsyjtx2025@163.com (G.L.); vbojiang2007@163.com (Y.J.); yubohao2005@163.com (Y.H.); yuyanghaas@163.com (Y.Y.); dongwenjun0911@163.com (W.D.); 2 College of Resources and Environment, Northeast Agricultural University, Harbin 150030, China; cppc161@163.com; 3 Key Laboratory of Original Agro-Environmental Pollution Prevention and Control, Agro-Environmental Protection Institute, Ministry of Agriculture and Rural Affairs, Tianjin 300191, China; liuzhongqi508@163.com

**Keywords:** rice, cadmium, malic acid, essential element, genotype

## Abstract

Cadmium (Cd) contamination poses a serious threat to rice safety and productivity. This study investigated the potential of malic acid (MA), a key metabolic organic acid, to mitigate Cd toxicity and its genotype-dependent effects on cadmium uptake and essential element homeostasis in rice. Using hydroponic experiments with multiple genotypes, we found that MA application (0.5–1.5 mmol·L^−1^) significantly reduced Cd accumulation in both roots and shoots, with the most effective reduction (up to 68.0%) achieved at 1.5 mmol·L^−1^. Notably, genotype X24 was a low-Cd accumulator, while genotypes 20, 58, and 65 were high accumulators. Beyond Cd reduction, this study reveals the profound and genotype-specific modulation of nutrient homeostasis by MA, including consistent suppression of K and enhancement of Ca across genotypes, and highly divergent responses in Mg, Mn, Fe, and Zn accumulation. Furthermore, MA dramatically alleviated Cd-induced inhibition of root morphology, particularly in the high-Cd genotype 58, increasing root length and tip number by 42.8% and 57.8%, respectively. Our results provide novel insights into the genotype-dependent rebalancing of essential elements under MA amendment, highlighting the crucial role of genetic background in plant responses to organic acid treatments. These findings provide a mechanistic basis for developing MA-based foliar conditioners and genotype-specific strategies for managing Cd contamination in rice.

## 1. Introduction

Rice (*Oryza sativa* L.) is one of China’s staple food crops. In recent years, Cd contamination in rice has become increasingly severe [1]. Annual pollution affects approximately 12 million tons of grain, resulting in economic losses estimated at CNY 20 billion [2,3].

Cd enters rice grains through multiple pathways, including atmospheric deposition, sewage irrigation, sludge application, and heavy metal-enriched fertilizers [4]. Atmospheric deposition is the primary source of Cd pollution in industrial areas [5]. Industrial and mining activities emit large amounts of Cd-containing gases and dust into the atmosphere. These pollutants enter the soil or adhere to rice plants via gravity or rainfall, subsequently being absorbed by roots and leaves and transported to grains during the filling stage. The closer the rice fields are to Cd pollution sources, the higher the Cd content in the grains [6,7]. In some industrialized countries, atmospheric deposition contributes nearly 50% of the Cd found in rice [8]. In China, sewage irrigation represents another major Cd pollution source [9], with increasing volumes of untreated wastewater elevating Cd levels in paddies [10,11,12]. Although sludge improves soil quality, it can also introduce Cd and other heavy metals [13,14,15]. Furthermore, phosphate fertilizers—some containing up to 174 mg·kg^−1^ Cd—have become a significant source of soil Cd pollution after long-term application [16,17,18,19].

Cd enters rice roots via both active (carrier protein-mediated, energy-dependent symplastic transport) and passive (electrochemical potential-driven apoplastic transport) mechanisms [20]. Following uptake, a portion of Cd is immobilized in roots (e.g., bound to cell walls, organic acids, or proteins [21,22,23]), while the remainder is translocated via the xylem to shoots, stems, and panicles, and finally accumulates in grains during filling [20]. Under Cd stress, rice activates detoxification pathways to mitigate toxicity [21,22,23,24,25], though the efficiency of these mechanisms—and thus Cd accumulation capacity—varies significantly across genotypes [26]. For example, hybrid rice exhibits stronger Cd uptake and transport abilities than conventional rice [27], and differences in root absorption and xylem transport efficiency are key drivers of genotypic variation in grain Cd content [28,29,30,31]. Additionally, the phloem plays a critical role in Cd translocation to grains, with variability in phloem transport further contributing to genotypic differences in grain Cd levels [32].

Cd toxicity also disrupts various physiological processes in rice: it damages chloroplast ultrastructure, reduces photosynthetic rate, stomatal conductance [33], and transpiration [34], and accelerates leaf chlorosis. Notably, chlorophyll stability and the rate of chlorosis have been proposed as practical indicators for rapidly assessing Cd tolerance among rice varieties [35]. Beyond impairing crop productivity, Cd accumulated in rice grains enters the food chain, posing serious health risks to humans and animals [36,37,38,39]. These impacts highlight the urgency of developing effective strategies to reduce Cd accumulation in rice.

Malic acid (MA), a key small-molecule organic acid in the tricarboxylic acid (TCA) cycle, links metabolic processes across plant organelles and plays pivotal roles in plant stress adaptation [40]. Under stress conditions, plants produce and secrete organic acids to regulate rhizosphere cation exchange capacity, modulate nutrient balance, and enhance tolerance to toxic metals [41,42]. For heavy metals like Cd, organic acids can bind to Cd in the xylem, converting it into less toxic forms and alleviating translocation-related toxicity [43]. Exogenous MA has been shown to promote plant growth, increase net photosynthetic rate, reduce H_2_O_2_ accumulation, and improve root activity under Cd stress [44], while root-secreted MA is closely associated with Cd tolerance in rice. However, conflicting findings exist—some studies suggest root-secreted organic acids may mobilize soil Cd, increasing its bioavailability and promoting accumulation in certain plants [45,46].

Given rice’s high Cd enrichment capacity among staple cereals [47,48,49], and the need for effective, sustainable Cd mitigation strategies, this study aimed to (1) determine the optimal MA concentration for inhibiting Cd uptake and translocation in rice seedlings; (2) elucidate the genotype-dependent effects of MA on Cd accumulation; and (3) explore how MA modulates essential element homeostasis under Cd stress. The findings are expected to provide a mechanistic basis for developing MA-based foliar conditioners and genotype-specific Cd management strategies for rice in heavy-metal-polluted regions.

## 2. Materials and Methods

### 2.1. Experimental Materials

The experimental materials are rice germplasm resources preserved in the laboratory and rice genotypes grown on a large scale in the south of China (Table 1). All chemical reagents used in this study, unless otherwise specified, were of analytical grade and purchased from Sinopharm Chemical Reagent Co., Ltd. (Shanghai, China).

### 2.2. Hydroponic Culture Test

Uniform and healthy rice seeds were selected, sterilized with 5% sodium hypochlorite for 15 min, rinsed with deionized water, and placed in sterilized trays with deionized water covering the seeds. The trays were incubated at 30 °C in the dark for germination. After root and shoot emergence, seedlings were transferred to a climate chamber for light exposure. At the two-leaf stage, seedlings were moved to 8 L hydroponic boxes (100 seedlings per box) containing 1/10 Hoagland nutrient solution. This setup ensured that from the two-leaf stage onward, all seedlings were consistently maintained in the nutrient solution.

Treatments were initiated at the three-leaf stage. The experiment adopted a randomized complete block design (RCBD), with each 8 L hydroponic box serving as an independent experimental unit. All hydroponic boxes were randomly placed in the climate chamber to eliminate potential positional interference (e.g., uneven light or temperature distribution). The experiment comprised a control group (CK) and three treatment groups, each with three independent biological replicates (*n* = 3). The compositions were as follows: CK (no additions), M0 (0.3 mg·L^−1^ Cd), M0.5 (0.3 mg·L^−1^ Cd + 0.5 mmol·L^−1^ malic acid), and M1.5 (0.3 mg·L^−1^ Cd + 1.5 mmol·L^−1^ malic acid). Prior to treatment, seedlings from all groups underwent a 24 h pre-starvation in deionized water to standardize their nutritional status. Following this, the standard 1/10 Hoagland solution was applied and refreshed every two days for a 10-day treatment period. The pH of all solutions was adjusted to and maintained within the range of 5.5–6.0 throughout the experiment. The climate chamber conditions were light period 25 °C, 16 h, 40% humidity; dark period 20 °C, 8 h, 60% humidity. After treatment, rice seedling roots were immersed in 5 mmol·L^−1^ CaCl_2_ for 15 min to remove surface Cd^2+^, followed by three rinses with deionized water. Some seedlings were used for root scanning, while others were dried to constant weight for Cd and essential element analysis.

### 2.3. Determination of Root Morphology

Washed rice seedlings (*n* = 20) were scanned using a root scanner (Perfection V700 Photo, Seiko Epson Corp., Tokyo, Japan). The resulting images were analyzed with WinRHIZO software (version 2009a, Regent Instruments, Québec City, QC, Canada) [50] to measure total root length, root surface area, root volume, and root tip number.

### 2.4. Determination of Cd and Essential Elements

Rice root and shoot samples (0.5 g each) were digested with 7 mL HNO_3_, left at room temperature for 12 h, and heated at 110 °C for 2.5 h. After cooling, 1 mL H_2_O_2_ was added, and heating continued for 1.5 h. The digest was evaporated to <1 mL at 170 °C, diluted, and analyzed for Cd, K, Ca, Mg, Fe, Mn, and Zn using ICP-MS (NexION^®^1000, PerkinElmer, Waltham, MA, USA) at Nanjing Webiolotech Testing Technology Co., Ltd. (Nanjing, China).

### 2.5. Data Processing and Calculation Method

Data were analyzed using IBM SPSS statistics (version 26.0). Significant differences among treatments were determined by Duncan’s multiple range test at *p* < 0.05 level. All data are presented as mean ± standard deviation (SD) of three biological replicates. Figures were prepared using Origin 8.6 (version 8.6).

## 3. Results

### 3.1. Genotypic Differences in Cadmium Accumulation in Rice

**Figure 1:**
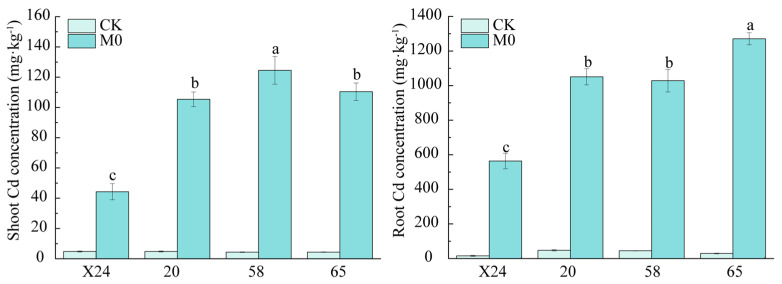
Cadmium concentration in rice root and shoot under 0.3 mg·L^−1^ Cd exposure. CK: Untreated control group (no additions); M0: 0.3 mg·L^−1^ Cd (sole Cd stress treatment). Different letters indicate a significant difference between different genotypes at 5% level.

Rice seedlings of genotypes X24, 20, 58, and 65 were subjected to 0.3 mg·L^−1^ Cd stress for ten days, and significant differences in Cd accumulation in shoots and roots were observed among the rice genotypes (Figure 1). Among the four genotypes, X24 was identified as a low-Cd-accumulating variety, with significantly lower Cd concentrations in both shoots and roots compared to genotypes 58 and 65 (Figure 1). Genotype 58 showed the highest shoot Cd accumulation, while genotype 65 had the highest root Cd accumulation, consistent with their classification as high-Cd accumulators. In summary, under 0.3 mg·L^−1^ Cd stress, genotypes 58 and 65 showed slightly higher Cd accumulation capacity than genotype 20, which in turn accumulated more Cd than genotype X24.

### 3.2. Effects of Cd on Essential Element Accumulation in Rice Genotypes

To explore how Cd stress interacts with genotypic differences to affect nutrient uptake, we analyzed the accumulation of essential elements (K, Ca, Mg, Fe, Mn, Zn) in roots and shoots of the four genotypes under Cd-free and Cd-stressed conditions.

**Figure 2:**
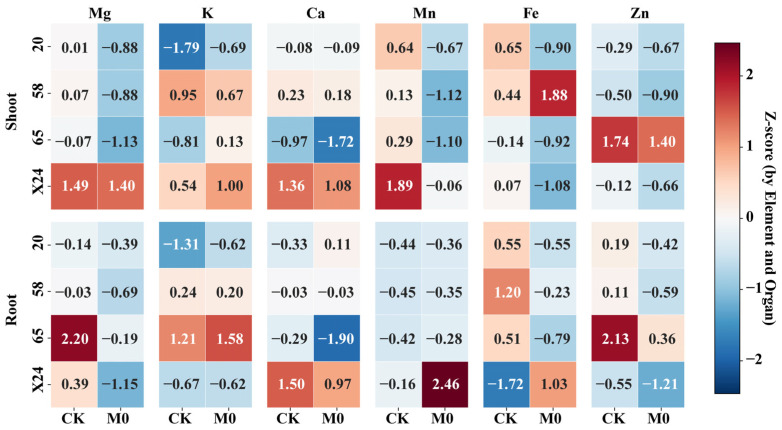
Heatmap of Z-scores for essential element accumulation in roots and shoots of four rice genotypes under Cd and control conditions. CK: Untreated control group (no additions); M0: 0.3 mg·L^−1^ Cd (sole Cd stress treatment).

Under the Cd-free condition, significant genotypic differences in essential element accumulation were observed among the four rice genotypes. In conditions without Cd stress, root Mg, K, and Zn accumulation was notably more prominent in genotype 65, while X24 demonstrated higher levels of Ca and Mn (Figure 2, Table A1). In the shoots, X24 maintained elevated Mg, Ca, and Mn content; genotype 58 had the highest K accumulation; and genotype 65 showed the greatest Zn accumulation. These results clearly reveal the distinct patterns of elemental accumulation among different genotypes (Figure 2). A similar pattern was observed in shoots: genotype X24 maintained higher accumulation of Mg (Z = 1.49), Ca (Z = 1.36), and Mn (Z = 1.89); genotype 58 had the highest K (Z = 0.95); genotype 65 showed the highest Zn (Z = 1.74); and genotype 20 displayed slightly higher Fe levels (Z = 0.65). These results indicate distinct elemental accumulation patterns among genotypes, with 65 showing strong Zn affinity and X24 exhibiting strength in multiple elements (Mg, Ca, Mn), demonstrating consistent root-shoot accumulation patterns.

Under Cd stress, both common and genotype-specific responses were observed (Figure 2, Table A2). Common responses included: significant reduction in root Mg and Zn (ΔZ < 0) and shoot Mg, Ca, Mn, and Zn (ΔZ < 0) across all genotypes; and increased root Mn accumulation (ΔZ > 0), although this effect was most pronounced in genotype X24 (ΔZ = +2.62). Genotype-specific responses revealed the following: Cd significantly enhanced root Fe accumulation only in X24 (ΔZ = +2.75) while reducing it in others; specifically promoted shoot Fe accumulation in genotype 58 (ΔZ = +1.44); and differentially affected K distribution, increasing root K in genotypes 20 and 65 but decreasing shoot K in genotype 58.

These findings demonstrate that Cd stress generally disrupts nutrient homeostasis but elicits genotype-specific adaptations, particularly in Fe and Mn accumulation, suggesting distinct genetic strategies for coping with cadmium toxicity.

### 3.3. Effects of Malic Acid on Cd Accumulation in Rice Genotypes

**Figure 3:**
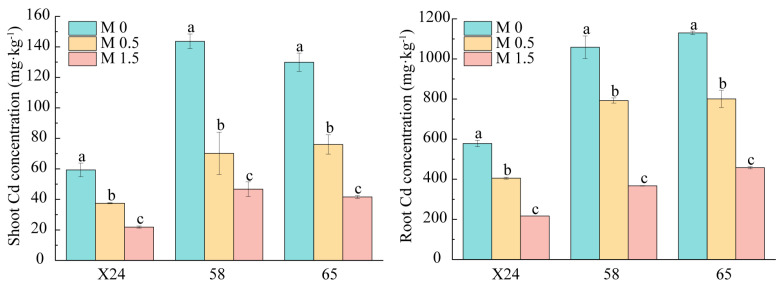
Effect of malic acid on cadmium accumulation and partitioning in rice root and shoot under Cd stress. M0: 0.3 mg·L^−1^ Cd (sole Cd stress treatment); M0.5: 0.3 mg·L^−1^ Cd + 0.5 mM malic acid; M1.5: 0.3 mg·L^−1^ Cd + 1.5 mM malic acid. Different letters indicate a significant difference between different genotypes at 5% level.

When rice seedlings of genotypes X24, 58, and 65 were exposed to 0.3 mg·L^−1^ Cd stress, with the addition of 0.5 or 1.5 mmol·L^−1^ malic acid, Cd accumulation in both shoots and roots significantly decreased, with varying degrees of reduction among genotypes (Figure 3).

Without malic acid, genotype X24 showed significantly lower Cd accumulation in shoots and roots compared to genotypes 58 and 65. Compared to the control (M0), 0.5 and 1.5 mmol·L^−1^ malic acid reduced shoot Cd content in genotype X24 by 36.8% and 63.2%, respectively, and root Cd content by 29.9% and 62.5%. For genotype 58, shoot Cd content decreased by 51.2% and 67.5%, and root Cd content by 25.1% and 65.3%. In genotype 65, shoot Cd content declined by 41.6% and 68.0%, and root Cd content by 29.2% and 59.5%. These results demonstrate that 0.5, 1.5 mmol·L^−1^ malic acid effectively inhibits Cd accumulation, with significant genotypic differences in sensitivity to malic acid.

### 3.4. Effects of Malic Acid on Essential Elements in Rice Genotypes

**Figure 4:**
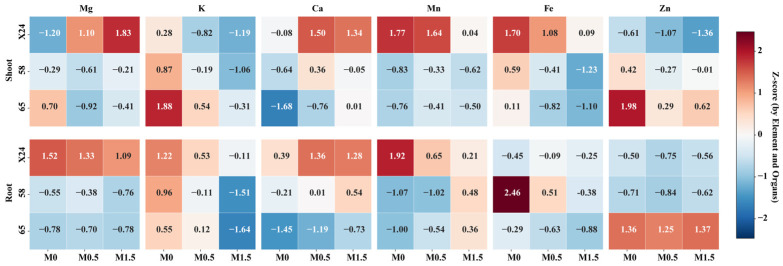
Heatmap of Z-scores for essential element accumulation in roots and shoots of rice genotypes X24, 58, and 65 under malic acid treatments. M0: 0.3 mg·L^−1^ Cd (sole Cd stress treatment); M0.5: 0.3 mg·L^−1^ Cd + 0.5 mM malic acid; M1.5: 0.3 mg·L^−1^ Cd + 1.5 mM malic acid.

The application of exogenous malic acid (0.5 and 1.5 mmol·L^−1^) induced pronounced, genotype-dependent shifts in the accumulation of essential elements in both roots and shoots (two key nutritional organs) of rice seedlings (Genotypes X24, 58, and 65), as detailed by Z-score analysis (Figure 4).

Root responses revealed both common and genotype-specific patterns across all three genotypes (Figure 4, Table A3). A consistent regulatory effect was observed for potassium (K) and calcium (Ca): malic acid significantly suppressed K accumulation and notably enhanced Ca accumulation in roots of all genotypes. Conversely, the effects on zinc (Zn), iron (Fe), magnesium (Mg), and manganese (Mn) were highly genotype-specific. In genotype X24, malic acid application maintained relatively high Mg accumulation (Z-scores: 1.52 → 1.33 → 1.09) and slightly reduced Mn accumulation (1.92 → 0.66 → 0.21), though Mn levels remained above the overall average (Z-score > 0). In genotype 58, a notable promotion of Mn accumulation was observed specifically under the 1.5 mmol·L^−1^ malic acid treatment (Z-score: −1.07 → 0.48, shifting from below to above average), while Fe accumulation was strongly suppressed (2.46 → −0.38). Genotype 65 exhibited a similar trend of Mn promotion (−1.00 → 0.36) and Fe suppression (−0.29 → −0.88), while maintaining stably high Zn levels (Z-scores > 1.24) across all malic acid treatments.

Shoot responses demonstrated even greater genotypic divergence compared to roots (Figure 4, Table A4). Most strikingly, in genotype X24, malic acid triggered a dramatic shift in Mg and Ca homeostasis: Mg accumulation increased from below the overall average to a highly abundant state (Z-score: −1.20 → 1.83), and Ca accumulation also significantly elevated (from −0.08 to 1.34). This shift was accompanied by a concurrent reduction in the accumulation of K, Fe, and Zn. The responses of genotypes 58 and 65 were markedly different from that of X24. Both genotypes showed increased Ca accumulation (e.g., genotype 65: Z-score −1.68 → 0.01) and decreased K accumulation in shoots; however, unlike X24, their Mg, Fe, and Zn status remained largely unchanged. Notably, Mn accumulation in shoots was consistently high in genotype X24 (Z-score > 0) but persistently low in genotypes 58 and 65 (Z-score < 0) across all treatments, highlighting a fundamental genotypic difference in Mn translocation and accumulation.

In summary, exogenous malic acid consistently modulates the distribution of K and Ca in both roots and shoots across the three rice genotypes, while eliciting highly divergent, genotype-specific effects on the homeostasis of Mg, Mn, Fe, and Zn. These findings underscore that genetic background is a primary determinant of the nutritional responses of rice seedlings to organic acid amendments.

### 3.5. Effect of Malic Acid on Root Morphology of Rice Genotypes Under Cd Stress

This study selected two rice genotypes (58 and 65) for investigation, which were treated with 0.3 mg·L^−1^ Cd for 10 days. The rice root system was first affected by Cd toxicity, and the root length, root surface area, root volume, and root tip number of seedlings could reflect the tolerance of rice root organs to Cd.

**Figure 5:**
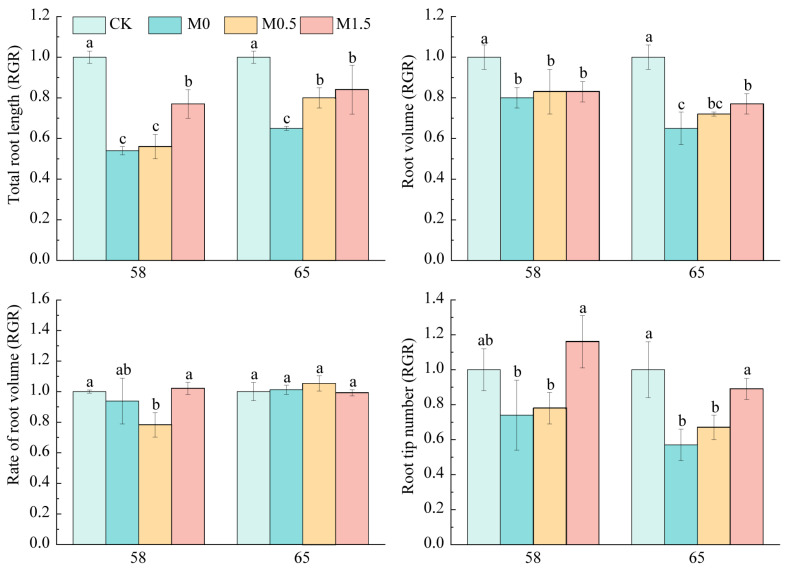
Malic acid rescues cadmium-impaired root growth across rice genotypes and doses. RGR (Relative Growth Rate) was calculated as the ratio of [trait value in treatment] to [trait value in control]. Values > 1 indicate growth promotion, while values < 1 indicate growth inhibition. CK: Untreated control group (no additions); M0: 0.3 mg·L^−1^ Cd (sole Cd stress treatment); M0.5: 0.3 mg·L^−1^ Cd + 0.5 mM malic acid; M1.5: 0.3 mg·L^−1^ Cd + 1.5 mM malic acid. Different letters indicate a significant difference between different genotypes at 5% level.

Compared with the CK treatment, 0.3 mg·L^−1^ Cd significantly inhibited root development in rice genotype 58, reducing root length by 45.8%, root surface area by 19.8%, root volume by 6.2%, and root tip number by 26.4%. Notably, the reductions in root length and surface area reached statistical significance (*p* < 0.05, Figure 5). The addition of 0.5 and 1.5 mmol·L^−1^ malic acid effectively mitigated these Cd-induced inhibitory effects. Compared with the M0 treatment, 1.5 mmol·L^−1^ malic acid treatment resulted in significant increases of 42.8% in root length and 57.8% in root tip number (Figure 5).

Under 0.3 mg·L^−1^ Cd stress, the root length, root surface area, and root tip number of genotype 65 decreased significantly by 35.1%, 35.2%, and 42.6%, respectively, compared with CK treatment. Adding 0.5 and 1.5 mmol·L^−1^ malic acid could alleviate the inhibitory effect of Cd on the root length, root surface area, and root tip number of genotype 65 seedlings. Compared with the M0 treatment, 1.5 mmol·L^−1^ malic acid could significantly increase the root length, root surface area, and root tip number by 28.9%, 18.0%, and 55.4%. In summary, Cd inhibits root growth in both genotype 58 and 65, manifesting as a decrease in root length and root surface area parameter values and a decrease in root tip number.

Adding malic acid can significantly reduce the inhibitory effect of Cd on root development. It is speculated that adding 0.5~1.5 mmol·L^−1^ malic acid to the root system can promote the growth and development of rice roots, reduce the toxic effect of Cd on rice roots, and have a positive effect on the entire growth period of rice.

## 4. Discussion

**Figure 6:**
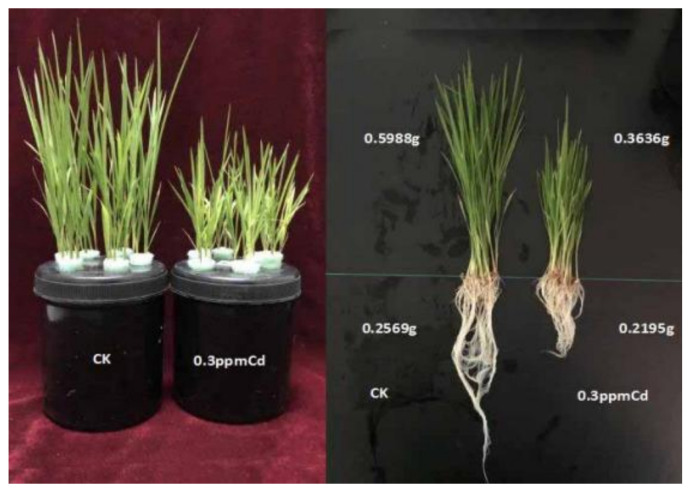
Effects of Cd on the growth of rice seedlings. CK: Untreated control group (no additions); 0.3 ppm Cd: 0.3 mg·L^−1^ Cd (sole Cd stress treatment).

Cadmium, as a toxic heavy metal, significantly inhibits plant growth and development. In our study, exposure to 0.3 mg·L^−1^ Cd (the concentration used in this experiment) significantly inhibited the growth of rice seedlings, as evidenced by reduced seedling fresh weight (Figure 6). This aligns with previous findings that Cd toxicity retards rice growth at low concentrations and severely impairs all growth stages or even causes mortality at high concentrations [45,46]. Cd influences malic acid secretion in rice roots, with low concentrations promoting and high concentrations inhibiting its production [51]. While some studies suggest that root-secreted organic acids can activate Cd in the rhizosphere and enhance its bioavailability [52,53,54,55,56], others report that exogenous malic acid does not promote Cd uptake [57,58], indicating complex interactions between organic acids and Cd accumulation in rice [59].

Our comparative analysis revealed distinct genotypic patterns in Cd accumulation: genotype X24 consistently showed low Cd accumulation, whereas genotypes 20, 58, and 65 were high accumulators (Figure 1). Notably, the addition of 0.5 or 1.5 mmol·L^−1^ malic acid significantly reduced Cd accumulation in roots and shoots of genotypes X24, 58, and 65 under Cd stress, with optimal reduction at 1.5 mmol·L^−1^ (Figure 3). This protective effect may be attributed to malic acid’s role in critical metabolic processes that inhibit Cd bioavailability and absorption [60].

A central finding of this study is that genetic background fundamentally determines how rice genotypes respond to both Cd stress and malic acid amendment, particularly regarding essential element homeostasis. While Cd is known to interfere with mineral nutrient uptake [61]—suppressing Mn and Zn in wheat roots [62] while promoting P, K, and Mn absorption [63]—our standardized Z-score analysis revealed not only genotypic variation in inhibitory effects but also a clear distinction between common stress responses and genotype-specific adaptations. Under Cd-free conditions, genotype 65 exhibited superior Zn accumulation capacity (Z = 2.13 in roots, Z = 1.74 in shoots), while genotype X24 showed prominent accumulation of multiple elements (Mg, Ca, and Mn) with consistent root–shoot distribution patterns. Under Cd stress, all genotypes showed reduced root Mg/Zn and shoot Mg/Ca/Mn/Zn (ΔZ < 0), yet only genotype X24 displayed increased root Fe (ΔZ = +2.75) and substantial root Mn enhancement (ΔZ = +2.62), suggesting Mn absorption may be a key adaptive trait for X24 in coping with Cd toxicity.

Most significantly, our results demonstrate that malic acid’s effect on nutrient homeostasis is profoundly genotype-dependent. Under 0.3 mg·L^−1^ Cd stress, malic acid consistently suppressed K accumulation and enhanced Ca accumulation across all genotypes—a common regulatory pattern potentially linked to conserved physiological roles in stress response. However, for other elements, responses varied dramatically by genotype: malic acid maintained high Mg and moderately reduced Mn (though still above average) in X24 roots; promoted Mn accumulation while strongly suppressing Fe in genotype 58; and enhanced Mn while maintaining high Zn levels in genotype 65. This genotypic divergence underscores that genetic background is a primary determinant of nutritional responses to organic acid amendments.

The mechanistic basis for these responses may involve multiple pathways. Previous studies have suggested that organic acids such as malic acid can form complexes with Cd, reducing its bioavailability, or compete for metal ion transporters responsible for Cd^2+^ uptake. The consistent reduction in K accumulation alongside Fe and Zn suppression suggests that malic acid may modulate non-selective cation channels [64] through which these elements and Cd enter rice cells, thereby reducing Cd transport while independently enhancing Ca uptake via specific transporters. Cd’s physicochemical similarity to essential elements like K, Fe, and Zn enables it to compete for binding sites on carrier proteins [65], further complicating these interactions.

We observed striking genotypic divergence in shoot responses, particularly regarding Mg and Mn homeostasis. Malic acid triggered a dramatic shift in Mg and Ca accumulation specifically in genotype X24 shoots (Mg: Z = −1.20 → 1.83; Ca: Z = −0.08 → 1.34), while genotypes 58 and 65 showed minimal changes in these elements. Furthermore, Mn accumulation remained consistently high in X24 shoots but persistently low in 58 and 65 across all treatments, highlighting fundamental genotypic differences in Mn translocation efficiency.

The uniform increase in Ca accumulation across genotypes has important physiological implications for Cd stress mitigation. As calcium pectate in the cell wall matrix, enhanced Ca likely contributes to improved cell wall formation and intracellular osmotic regulation, maintaining normal physiological conditions under stress. Additionally, Ca’s involvement in protein synthesis and enzymatic metabolism further supports plant growth and development under Cd stress. This mechanism may explain why malic acid significantly alleviated Cd-induced root growth inhibition in genotypes 58 and 65 (Figure 3), possibly through its participation in diverse metabolic pathways and enzyme formation that stimulate meristematic cell proliferation.

In conclusion, our findings demonstrate that 1.5 mmol·L^−1^ malic acid not only promotes root development and mitigates Cd toxicity but also modulates essential element homeostasis in a fundamentally genotype-dependent manner. The genetic background of the host plant emerges as a primary factor determining whether malic acid amendment will suppress, promote, or have minimal effect on specific essential elements, providing crucial insights for developing genotype-specific strategies for heavy metal stress management in rice. While our physiological and elemental data strongly support a role for malic acid in modulating Cd uptake and nutrient homeostasis, future studies incorporating gene expression analyses and direct measurement of metal–organic acid complexes will be essential to fully elucidate the underlying molecular mechanisms.

## 5. Conclusions

Malic acid serves as an effective amendment for mitigating cadmium toxicity in rice, but its efficacy and mechanistic actions are fundamentally governed by the plant’s genetic background. We identified distinct Cd accumulation patterns among genotypes, with X24 exhibiting low accumulation and 58/65 being high accumulators. The key finding is that 1.5 mmol·L^−1^ MA optimally reduced Cd accumulation across these genotypes. More importantly, this study unveils a novel, genotype-specific reprogramming of essential element homeostasis induced by MA, characterized by consistent suppression of K and promotion of Ca, alongside highly divergent responses in Mg, Fe, Mn, and Zn accumulation. This genotypic variation extends to physiological responses, as MA significantly restored Cd-impaired root growth in sensitive genotypes. The dual role of MA in reducing Cd uptake and modulating nutrient equilibrium represents a significant advancement over previous studies focused solely on Cd mitigation. Therefore, the application of MA represents a promising strategy for reducing Cd bioavailability and accumulation in rice. However, given the strong genotype dependence, we emphasize the necessity of considering genetic profiles in developing MA-based agronomic practices or foliar conditioners, which constitutes a novel contribution to the field.

## Figures and Tables

**Table 1:** List of different rice materials.

List	Genotype
X24	Xiang zao shan 24
20	Nan xiong zao you zhan
58	Zhu zhen B
65	Zhen shan 97B

## Data Availability

All data generated or analyzed during this study are included in this published article.

## Abbreviations

The following abbreviations are used in this manuscript:

Cd	Cadmium
TCA	Tricarboxylic acid
CK	Control group
MA	Malic acid

## Appendix A

**Table A1:** The accumulation of essential elements in rice roots and shoots of different genotypes (Mean ± SD, mg·kg^−1^). Different letters indicate a significant difference between different genotypes at 5% level.

	Genotype	Mg	K	Ca	Mn	Fe	Zn
Root	X24	1122 ± 280 bc	12,358 ± 2461 a	3480 ± 220 a	128 ± 48 a	2316 ± 146 b	205 ± 47 b
	20	987 ± 76 c	9681 ± 1324 d	2555 ± 198 b	45 ± 11 b	4998 ± 126 a	291 ± 32 b
	58	1014 ± 79 bc	16,099 ± 778 b	2707 ± 107 b	43 ± 3 b	5757 ± 315 a	281 ± 13 b
	65	1586 ± 46 a	20,130 ± 592 a	2579 ± 148 b	50 ± 8 b	4951 ± 289 a	514 ± 47 a
Shoot	X24	6061 ± 414 a	52,747 ± 1729 a	6463 ± 479 a	676 ± 33 a	369 ± 138 b	131 ± 16 b
	20	4842 ± 60 b	45,501.7 ± 984 b	5382 ± 166 bc	469 ± 23 b	454 ± 65 a	119 ± 8 b
	58	4893 ± 439 b	54,039 ± 6137 a	5616 ± 340 b	384 ± 25 c	424 ± 101 a	104 ± 14 b
	65	4775 ± 180 b	48,533 ± 2423 b	4717 ± 141 c	411 ± 16 c	338 ± 54 b	263 ± 6 a

**Table A2:** The content of essential elements in rice roots and shoots of different genotypes under 0.3 mg·L^−1^ Cd stress (Mean ± SD, mg·kg^−1^). Different letters indicate a significant difference between different genotypes at 5% level.

	Genotype	Mg	K	Ca	Mn	Fe	Zn
Root	X24	728 ± 22 d	12,568 ± 1698 c	3214 ± 157 a	907 ± 106 a	5562 ± 2089 a	129 ± 13 d
	20	922 ± 20 b	12,543 ± 257 c	2779 ± 43 b	70 ± 2 b	3700 ± 73 ab	220 ± 11 b
	58	846 ± 26 c	15,955 ± 776 b	2709 ± 61 b	71 ± 6 b	4073 ± 257 ab	201 ± 10 c
	65	975 ± 41 a	21,667 ± 1095 a	1766 ± 190 c	92 ± 41 b	3422 ± 155 b	310 ± 7 a
Shoot	X24	5987 ± 112 a	54,199 ± 885 a	6255 ± 52 a	353 ± 8 a	201 ± 44 b	93 ± 15 b
	20	4107 ± 330 b	48,909 ± 3883 a	5375 ± 450 b	252 ± 22 b	227 ± 24 b	92 ± 9 b
	58	4114 ± 301 b	53,145 ± 3525 a	5574 ± 272 b	178 ± 11 c	634 ± 335 a	76 ± 4 b
	65	3905 ± 140 b	51,482 ± 490 a	4155 ± 111 c	182 ± 15 c	225 ± 121 b	239 ± 6 a

**Table A3:** Effect of malic acid on the accumulation of essential elements in rice roots of different genotypes (Mean ± SD, mg·kg^−1^). Different letters indicate a significant difference between different genotypes at 5% level.

	Genotype	Mg	K	Ca	Mn	Fe	Zn
X24	M0	728 ± 22 c	12,568 ± 1698 a	3214 ± 157 b	907 ± 106 a	5562 ± 2089 a	129 ± 13 a
	M0.5	1027 ± 52 b	6317 ± 2087 b	4634 ± 83 a	863 ± 312 a	4721 ± 369 a	97 ± 12 b
	M1.5	1121 ± 59 a	4264 ± 555 b	4487 ± 71 a	351 ± 12 b	3403 ± 1138 a	77 ± 11 b
58	M0	846 ± 26 a	15,955 ± 776 a	2709 ± 61 b	71 ± 6 c	4073 ± 257 a	201 ± 10 a
	M0.5	805 ± 59 a	9915 ± 699 b	3602 ± 672 a	230 ± 13 a	2727 ± 226 b	153 ± 11 b
	M1.5	856 ± 26 a	4974 ± 250 c	3235 ± 144 ab	139 ± 7 b	1628 ± 58 c	171 ± 25 ab
65	M0	975 ± 41 a	21,667 ± 1095 a	1766 ± 190 c	92 ± 4 c	3422 ± 155 a	310 ± 7 a
	M0.5	765 ± 61 b	14,047 ± 715 b	2596 ± 19 b	205 ± 7 a	2182 ± 98 b	192 ± 5 c
	M1.5	831 ± 52 b	9259 ± 919 c	3292 ± 167 a	176 ± 10 b	1806 ± 34 c	215 ± 4 b

**Table A4:** Effect of malic acid on the accumulation of essential elements in rice shoots of different genotypes (Mean ± SD, mg·kg^−1^). Different letters indicate a significant difference between different genotypes at 5% level.

	Genotype	Mg	K	Ca	Mn	Fe	Zn
X24	M0	5987 ± 112 a	54,199 ± 885 a	6255 ± 52 a	353 ± 8a	201 ± 44a	93 ± 15 a
	M0.5	5816 ± 409 a	51,404 ± 3270 ab	7363 ± 841 a	279 ± 27 b	255 ± 106 a	73 ± 8 a
	M1.5	5602 ± 393 a	48,853 ± 2872 b	7276 ± 609 a	253 ± 7 b	231 ± 126 a	88 ± 6 a
58	M0	4113 ± 301 a	53,145 ± 3525 a	5574 ± 272 b	178 ± 11 b	634 ± 335 a	76 ± 4 a
	M0.5	4267 ± 32 a	48,831 ± 572 b	5820 ± 54 b	181 ± 3 b	343 ± 6 ab	66 ± 1 b
	M1.5	3923 ± 21 a	43,179 ± 968 c	6427 ± 171 a	269 ± 3 a	211 ± 20 b	83 ± 5 a
65	M0	3905 ± 140 a	51,482 ± 490 a	4155 ± 111 b	182 ± 15 b	225 ± 121 a	239 ± 6 a
	M0.5	3980 ± 318 a	49,770 ± 4399 a	4453 ± 381 b	209 ± 16 b	174 ± 56 a	230 ± 16 a
	M1.5	3903 ± 121 a	42,647 ± 1177 b	4977 ± 169 a	262 ± 18 a	138 ± 33 a	240 ± 19 a
